# A Lacanian view on Balint group meetings: a qualitative analysis of two case presentations

**DOI:** 10.1186/1471-2296-15-49

**Published:** 2014-03-21

**Authors:** Kaatje Van Roy, Stijn Vanheule, Virginie Debaere, Ruth Inslegers, Reitske Meganck, Julie Deganck

**Affiliations:** 1 Department of Psychoanalysis and Clinical Consulting, Ghent University, H. Dunantlaan 2, 9000 Ghent, Belgium

**Keywords:** Balint group, Qualitative research, Subject, Lacan, General practitioner

## Abstract

**Background:**

GPs’ subjectivity is an intrinsic instrument in their daily work. By offering GPs a platform to present and discuss difficult interactions with patients, Balint group work be might provide them an opportunity to explore and articulate aspects of their subjectivity. In order to get a more profound understanding of what participation in a Balint group can offer, we focused on the process of change that can be observed during Balint group meetings. To that end, this study scrutinized two Balint group case discussions on a micro-level.

**Method:**

Two cases were selected from a larger data set of 68 audio-taped case discussions in four Balint groups. In order to shed light on the type of change that characterizes the presenter’s narrative, we used Lacan’s theoretical distinction between imaginary and symbolic modes of relating to the other.

**Results:**

In both case discussions, the GPs presenting the case initially appeared to be stuck in a fixed image of a situation, referred to as ‘imaginary relating to the other.’ Through a range of interactions with the group, the presenters were encouraged to explore different subject positions, which allowed them to broaden their initial image of the situation and to discover other issues at stake. This was referred to as a more symbolic way of relating to the other.

**Conclusion:**

This study throws light on the type of change Balint group participation allows for and on the way this might be achieved. We conclude that Balint group work is potentially beneficial to the participating GPs as well as to the relationship with their patients.

## Background

While guidelines increasingly assist general practitioners (GPs) in making decisions with regard to medical diagnosis and treatment, less attention is given to their subjective experience and interpretation of clinical situations. Nevertheless, it is said that GPs “have to make decisions about what to say, what to treat, what to ignore, what to observe, what to reflect about and what to turn their backs on” (1979: 470) [1]. Consequently, apart from a vast amount of medical knowledge and technical expertise, they also use *themselves* as instruments in diagnosis and therapy [2]. In order to use themselves more effectively in their work, Novack et al. [2] suggest that physicians should “calibrate their instruments,” i.e. their own subjectivity. Among other methods of work-related self-reflection [2], Balint group work provides physicians with opportunities to explore and articulate their own subjective involvement in their everyday work [3,4].

Balint groups were first set up in the 1950s in London by the psychoanalyst Michael Balint [3-5]. These groups were designed to offer GPs a platform to explore difficult interactions with patients by means of case presentations and discussions. Since that time, Balint groups have been set up worldwide, albeit on a small scale [6]. Some groups are exclusively for GPs, whereas others also welcome other professionals from the (para) medical field (e.g. [7-10]). Typically, Balint groups comprise six to twelve participants and one or two leaders (also referred to as animators); meetings usually take place on a once- or twice-monthly basis over several years. The meetings start with a participant’s case presentation, which generally reflects a difficult interaction he/she has had with a patient. The case presentation is then followed by a group discussion that focuses on the thoughts, emotions and subjective reactions that the presentation evokes [11,12]. Generally, in one meeting, two cases are presented and discussed. Balint group meetings aim to stimulate a process akin to psychoanalytic ‘free association.’ Therefore, participants are asked to present cases without using notes or case files [3] and all group members are encouraged to share their ideas, associations, images and emotions evoked during the discussion. This way of working facilitates alternative viewpoints that may redefine the initial problem. Moreover, by speaking freely, members can become aware of their unconscious attitudes towards the patient or the situation in a way that helps them recognise their own implication.

Research on Balint groups is relatively scarce. Only a limited number of studies examine the actual process of Balint group case presentations and discussions (e.g. [8,10,13,14]). Whereas Michael Balint believed that long-term participation in such groups could lead to “a limited, though considerable change in the doctor’s personality” (1964: 299) [3], it remains unclear as to what kind of change takes place in the mind-set of clinicians who participate in these groups. In the present study, we examine the potential benefit of Balint group work by exploring the process of change on a micro-level. Through a detailed examination of two Balint group case discussions, we study the change that takes place in group members’ perspectives. Therefore, we use Jacques Lacan’s theoretical distinction between imaginary and symbolic modes of relating to the other.

### Lacan’s theory on imaginary and symbolic relating to the other

Jacques Lacan (1901–1981) was a French psychoanalyst who re-examined Sigmund Freud’s work, bringing it into dialogue with linguistics, mathematics, structuralism and other disciplines [15]. Given the fact that subjectivity, discourse and the unconscious are central concepts in Lacan’s theory, it was deemed an excellent reference frame for this study’s purpose. More specifically, we used Lacan’s distinction between imaginary and symbolic modes of relating to the other to guide us in analyzing the data. Lacan [16] discusses the roots of this *imaginary relation* in his theory of the mirror stage. This theory states that early in life, due to a lack of sensory and motor coordination and the primitive organization of libidinal life, the infant’s self-experience is fragmented, and only gradually becomes organized through the recognition of a self-image in the outside world. By means of ‘mirroring’, i.e. discerning self-images or images of others as mirror images, the child identifies with a body image that it regards as its own [17]. For Lacan, the mirror phase coincides with the inauguration of the *ego*. This type of identification is not restricted to infancy, but is continues throughout one’s life [18]. Imaginary functioning is efficient in that it allows people to understand each other. For instance, when we are ill and decide to consult a doctor, we identify with the role of patient. In this context, the doctor functions as a mirror in which we see ourselves as a patient. In other words, the patient needs the doctor in order to assume his role as a patient, and vice versa. This implies that human beings do not so much acquire an identity by assuming certain characteristics, but by ascribing characteristics to someone else and by positioning themselves in relation to such characteristics [19]. It is indeed in the interaction with others that identity is developed [20]. As mentioned above, the ego provides us with a sense of unity. However, this feeling is “an illusion that blinds us to what does not fit the image” (2009: 396) [17] and at times favours a one-dimensional view of situations. Moreover, in imaginary relations, everything can be played out in terms of the opposition: *same or different*[21], which possibly results in power struggles.

While imaginary identification has an organizing role in mental life, Lacan [16,22,23] stresses its accompanying tendency for misrecognition: it masks the heterogeneity of the subject through sustaining a sense of self-unity [16]. The *symbolic relation*, by contrast, starts from recognizing the otherness of the other (i.e. ‘the other does not coincide with the image I have of him/her’), as well as one’s own dividedness (i.e. the subject is divided across different identifications). These characteristics distinguish the *subject* from the *ego*. From a Lacanian point of view, the subject is an effect of the fact that we speak; it is “multiple, contradictory and not entirely rational” (2005: 76) [24]. As a result, subjectivity is “seen as complex, distributed and fragmented, permeated by social and discursive processes, yet intimately personal, as the subject invests these processes with desire and turns them to the very stuff of his or her being” (2009: 655) [25]. The symbolic relation implies an openness for exploring and naming the *multiplicity* that characterizes the subject-dimensions or subject positions [26]. The underlying idea is that repressing the subject eventually results in symptomatic behaviours and complaints, as well as in problems at the level of imaginary functioning (e.g. power struggles).

To our knowledge, Lacanian theory has not yet been applied to an analysis of Balint group functioning. However, we believe that using the theoretical framework outlined above can offer new insight. Given the centrality of both speech and social interaction in Balint groups, focusing on imaginary and symbolic relations can help us depicting the process of change that takes place in Balint group discussions. Indeed, problems brought forward in these discussions are often examples of how a GP has become stuck in a fixed image of a situation (see also [27]). As outlined below, the change induced in Balint group discussions often coincides with a change in perspective from ego to subject, paving the way for a symbolic rather than an imaginary mode of relating to the other.

## Methods

### Procedure

The data used in this study are part of a larger data set gathered in the context of a PhD project on GPs’ experiences with their practice. For this larger data set, the first author, a female researcher with a degree in medicine and psychology, observed monthly meetings of four Balint groups over a 15-month period (April 2011 – June 2012). In total, 45 meetings (87 case discussions) were observed; from these, 33 meetings (68 case discussions) were audio-recorded. Three groups were located in Wallonia, the French-speaking region of Belgium, and one in the Netherlands. In two groups all participants were GPs; the other two groups were mixed (including GPs, physiotherapists and nurses).

Following each Balint group meeting, the observer noted down descriptions of the case presentations as well as reflections on the dynamics of the group discussion. From these observations, we noticed that many meetings were characterized by a marked ‘change’ in the presenter’s discourse on the presented doctor-patient situation. In order to further examine the observed process of change, two audio-taped case presentations were selected from the larger dataset and were transcribed verbatim. Both cases were considered typical and thus representative for the majority of the observed meetings. Moreover, the second case was considered highly instructive due to the marked change in the presenter’s discourse during the case discussion as well as the remarkably positive case follow-up. Transcripts were studied by the six members of our research team (KV, SV, VD, RI, RM and JD), all clinical psychologists. It was agreed upon that in both cases, the presenter’s discourse changed substantially throughout the respective sessions. This study was approved by the Ghent University Committee for Medical Ethics.

### Participants and sample

The selected presentations were selected from two relatively similar Balint groups. Each group met once a month in meetings lasting between two and two and a half hours; they both had eight to ten participants; both groups were gender-mixed and the members’ mean age was 46 years in one group and 52 in the other. Whereas in one group all participants were GPs, the other group also comprised other professionals, such as nurses and physiotherapists. The mean number of years of participation in these Balint groups was approximately 4,5 years (range 1 to 10 years). Both groups were led by two animators, who were GPs or psychologists with a training in psychoanalysis. The presenters of the cases below were both female GPs, who had been participating in their respective Balint groups for several years.

### Data analysis

The data-analysis consisted of two major parts. In the first phase, we coded the transcripts inductively, remaining very close to the participants’ words. The transcripts were first subdivided into fragments, each covering a different idea that was brought up in the Balint group meeting. At the same time, this allowed us to mark turning points in the discussion. Later, the ideas were categorized in broader themes that each reflected a different focus on the difficulty that was presented: focus on patient as a person, focus on patient’s situation, focus on GP and focus on doctor-patient interaction. Apart from a first analysis of the content, we also coded the group interventions (e.g. ‘challenging presenter’s expression’, ‘informative question’, ‘providing opinion’, ‘introducing new perspective’). The authors first studied the transcripts separately and subsequently consulted with each other to discuss the patterns of change that appeared in the data. As patterns of change were discussed, it was decided to make use of Lacan’s theoretical distinction between symbolic and imaginary relations. Applying this conceptual framework to the data, we started the second part of the data-analysis. By identifying the switches from imaginary to symbolic relating to the other, and by analyzing the group interventions that were associated with these, an overarching idea on the kind of change Balint group discussions provoked in the mind-set of the clinician came to the fore. More specifically, this part of the analysis was performed with two main focuses. On the one hand, it was guided by a continual reflection on the position each presenter is speaking from and the position that is attributed to the other, i.e. the patient. On the other hand, we focused on the language used by each presenter. We mapped the evolutions in the subject positions expressed by each presenter, as well as the group interventions that contributed to these evolutions.

## Results

### Case 1 – ‘The dismissed shock absorber’

In response to the animator’s routine question as to *who would like to present a case*, one female GP was keen to present a situation. She reminded the group that she had wanted to present this case in the previous meeting and stated: “Well, and I still have this situation, with new developments because I am dismissed.” It should be mentioned that it was only later in the discussion that the meaning of this statement became clear to the other group members (i.e. the patient had ‘dismissed’ the GP). The group immediately agreed to hear more about this case, and the presenter went ahead:

“The first time I saw this lady, completely accidentally, she called me saying that she needed a doctor because she didn’t feel well. So, I arrive [at her place], she’s lying on a mattress in a room in a working-class house, and she’s obviously suffering from an anxiety attack. And so, I talk to her for a while and then, well, apparently, she thinks that she’ll have me as her doctor. You should know that this lady lived in that house, I mean apartment, that the apartment was rented by her companion of the moment, and that at that moment, there were three or two children in the apartment which had only two rooms....”.

These introductory phrases provide a good sketch of the presenter’s initial report of the case, which proves to be highly anecdotal and strongly focused on the patient’s complex and chaotic situation. This initial presentation illustrates how this GP was somewhat stuck in a restricted perception of the situation. On the one hand, her discourse predominantly focused on the patient and, in particular, the patient’s way of living; her ideas and questions on the role *she* played (i.e., the presenter’s difficulties and feelings) were, by contrast, left almost unmentioned. On the other hand, the abundance of details and anecdotal information contrasts with the scarcity of meta-reflection on the situation. The presenter frequently used passive formulations (e.g. “I am dismissed”; “she’ll have me as her doctor”), which reflect well her feelings of being overwhelmed by the situation.

After a while, one animator intervened by inviting the presenter to talk about *her own position* in the situation she just presented. Indeed, the presenter had not elucidated the reason (s) for presenting this case, nor had she formulated some kind of question towards the group. Clarifying this was found to be commonplace in most of the Balint group meetings that we observed. The focus of such elucidation or question (e.g. whether on the patient’s problem or on the presenter’s own difficulty) can provide a first impression of the presenter’s perspective and acknowledgement of his or her subjective implication in the situation. In this presentation, such clarification was not spontaneously offered by the presenter. Moreover, she proved to have difficulties to react to the animator’s intervention, providing more anecdotal information about the patient instead. Throughout the discussion, group members made numerous attempts to encourage the presenter to express her reasons for presenting this case, either through direct questioning (e.g. “And how are you yourself situated in this story?”; “What is bothering you?”) or suggestions (e.g. “I don't know what your question is, but I want to say, I have some difficulties with therapeutic ruptures”; “Maybe this [feeling of it being a tough situation] is the reason why she presented the case”). The presenter’s reactions to these questions and suggestions further illustrates how she is somewhat absorbed in the situation and has difficulties verbalising her subjective position (e.g. “*it* has always been a complex situation”, “*it* really deteriorated”, “I wanted to know whether you can provide me with some ideas about how I could have avoided *being taken in by* that inextricable situation”).

The group members’ interventions consisted of a mix of questions and invitations for reflection on the one hand, and of ideas and suggestions that open up additional perspectives on the case on the other hand. Some interventions, for instance, aimed to stimulate the presenter’s reflection on the doctor-patient interaction. For example, when a group member posited that they must have had some kind of bond during all those years, the presenter reported how she had been communicating with the patient by means of a notebook for some time, and the difficulties this eventually evoked for the patient. Later in the discussion, one group member asked: “I was wondering how you relate to each other, like a woman accomplice to a woman, like a sister (…)? Well, in fact [this comes down to] how you imagine your relationship [with this patient] functions for her. Like a mother? Or like what?” Interestingly, these suggestions triggered a recollection in the presenter about the patient calling her a friend. She referred to a situation where this patient had asked her for money “as a friend.” Here, the presenter herself did not spontaneously explore the role the patient had attributed to her, yet the group picked-up on this, guiding and inviting the presenter to occupy a different position.

Other group interventions addressed the presenter’s tendency towards rationalisation as well as the scarcity of affective references. On the one hand, the group challenged the presenter’s propensity to rationalise situations by questioning the assumptions underlying her rationalisations. For instance, the presenter’s conviction that a medical centre is more structured than a private practice was repeatedly put into question by several group members. On the other hand, the group actively engaged in the affective dimension. By verbalising their own affective states, either in relation to the situation (e.g. “It's an impossible situation”; “It's lost from the beginning”), in relation to the patient (e.g. “I like her, I find her dynamic”), or in relation to the presenter (“I think you’ve come a long way with her”), the group actively introduced a supplementary range of subject positions. Some of these comments prompted the presenter to verbalise fragments of her own affective implication in the situation. For example, one group member’s comment that “she [the presenter] has done a lot for her [the patient]” makes the presenter claim “it’s true, I’m sure,” adding “too much” and “I didn’t protect myself enough.” This remark possibly indicates a subtle change in the presenter’s perception of the doctor-patient relationship: the presenter finally appears as someone who does not merely endure a situation, but as someone who actually has a choice with regard to how she can react to the situation.

The interactions outlined above reflect how members of this Balint group jointly created different perspectives on the situation that was presented: group members helped the presenter to transcend her immediate way of perceiving the situation and to explore it from other subject positions. For instance, this became apparent through a remarkable re-definition of the doctor-patient relationship. Whereas in talking about the doctor-patient relationship, the presenter repeatedly used expressions reflecting an employer-employee context (e.g. “I am dismissed”; “she’ll have me as her doctor”; “she fired me”; “she imposed a timetable”), one group member’s remark concerning the position a GP can occupy in such complicated cases led the presenter to reframe her position: “Maybe I was too much of a shock absorber.” The shift to a different semantic frame as well as the presenter’s active formulation of her own position may indicate her subjective position had been affected. However, other opportunities to articulate new subject positions were not taken up by the presenter. For example, when a group member commented on the fact that she had lent money to this patient, defining this as a boundary he would never cross, the presenter emphasized that she *only* did so with this patient. This statement prompted an animator to ask “But what does she evoke? What has she evoked that makes you say *I only did this with her*? (…) It is something very strong, isn’t it?”. While this reaction invited the presenter to elaborate on the way she is affected by this patient, she did not follow the animator’s prompt, but merely referred to what the patient needed the money for. This illustrates how the presenter only partly engaged in the acknowledgement of her subjective position in relation to the patient.

Apart from immediate alterations in the presenter’s discourse, another indication of the change that the group discussion evoked can be found in the case follow-up, which usually takes place during the next Balint group meeting. Although the presenter had no subsequent professional contact with the patient (the patient had ‘dismissed’ the GP), there had been a brief encounter which the presenter discussed with the group. On the one hand, she continued to engage in a rather unaffected and passive mode of storytelling. She commented upon a moment when she had seen the patient in the street, using phrases such as “I thought I was *immune*”, “*One* would like to have some news” and “I say to myself, well, *she* hasn’t contacted me yet.” On the other hand, she also attempted to verbalise how she felt when she met the patient in the street: “But I made the reflection…, I can’t explain exactly what the feeling was like, but it was not a pleasant one. Whereas I thought I was immune, I wasn’t. (…) Seeing her like that, I had a strange…, a malaise, I don’t know, really a malaise.” Moreover, referring to the fact that she is not in the position to solicit information about the patient from other professionals, she defined herself in more active terms (“I have detached myself from it”). Her hesitant search for a suitable expression (showing ambivalence and indeterminacy) and the additional focus on her own emotions indicate that the discussion had had an effect on the presenter’s perspective, helping her to transcend the imaginary mode of relating to the patient, in which she appeared to have been the passive victim of the other.

### Case 2 – ‘The escaping approacher’

Following an animator’s question as to whether anybody had a case to present, the group remained silent for a while. Finally one female GP stated: “I have a case.” After checking whether anybody else wanted to present a case, the animator passed the floor to this GP. She began with a brief description of the patient (an 80-year old widow living in a nursing home), followed by an account of their first meeting:

“And so, I go and meet her for the first time, and our first interaction was rather peculiar. I introduce myself, and immediately, things are complicated: I called her by her maiden name [upon which she objects:] ‘No, no, no (*screaming*), that’s not how I’m addressed, I’m called Mrs Blah Blah Blah.’ Moreover, it’s a long and hyper-complicated name. I say to her: ‘Alright, ok.’ [She goes on]: ‘For 40 years I’m Mrs Blah Blah Blah, and so, you should address me that way.’ Ok, alright. ‘Because, you know, I’m the daughter of a statesman, Mr Blah Blah Blah.’ Actually, she’s a patient from (*country*), who has been living here since she was married, so for a really long time. She was married to a statesman, or something like that, all of her grandchildren are politicians. Well, so I say to myself, it’s rather peculiar to talk to me like that, but, well, maybe she is somewhat confused. So then we started talking, but I thought it was peculiar because I found her a real snob, a real snob. Appearances are hyper-important [to her], she told me 40 times she was the daughter of a statesman.”

This fragment illustrates well this GP’s general style of reporting during her initial case presentation. Unlike the previously discussed case presentation, this one is clearly marked by affectivity. The presenter’s sense of irritation is tangible through the examples she used to describe the patient (e.g. the patient’s insistence on being called by her marital name), through her tone of voice as she mimicked the patient’s way of speaking, and through the feelings she expressed about the patient (e.g. “she irritates me”, “it’s unbearable”). In a number of the presenter’s comments, the seeds for conflict escalation within a predominantly imaginary mode of relating to the patient are apparent: her focus on the patient’s aggressive behaviour functions as a mirror in which her own irritation is reflected. However, the presenter also outlined various attempts to try to understand the patient’s behaviour (e.g. “Well, so I say to myself, it is rather peculiar to talk to me like that, but, well, maybe she is somewhat confused”). At first, these reflections all seem to revolve around her decision as to whether or not the patient suffers from ‘cognitive problems’ without taking into account other possible interpretations. The case presentation ended with the presenter narrating her attempts to go beyond the patient’s hostility by trying to engage her in different topics of conversation, attempts which proved to be vain. She concluded: “I have trouble relating to this patient,” “I don’t know what she is looking for” and “I can’t develop a rapport with her.”

One animator picked-up on these comments to open up the group discussion. A simple informative question (inquiring about the size of the patient’s room) led the presenter to state from a more reflective perspective that indeed the patient’s discourse did not tally with some of the actual facts (e.g. her family’s social standing versus the small room she’s living in). This incongruity was further elaborated by the group, portraying the patient’s situation as “past glory” and “a nineteenth century lady addressing her domestics” and suggesting the possibility that this patient might have been ‘fleeced’ by her children.

By explicitly designating the patient’s behaviour as *a role* she is taking up, one group member opened up further reflection on the meaning of this behaviour. Several dynamics were suggested: perhaps the patient feels humiliated and that is why she humiliates others; perhaps she is suffering and unable to admit it; the patient might be uprooted; “piquing” might keep her vivid; her behaviour might reflect resistance (against getting old, against her family that put her in the nursing home). In this part of the discussion, new perspectives were jointly constructed: several group members provided alternative ideas for understanding the patient, which were then commented upon by the presenter. One animator denominated these attempts to understand the patient as “a movement of compassion passing through the group.” The presenter then stated, with a notably softer voice: “I would like to approach her, but I have the impression that she won’t let me.” At this point in the discussion, the initial feelings of irritation towards the patient appeared to have been replaced by feelings of ‘compassion.’ On the one hand, this shift might be understood as transgressing the fixity of feelings of irritation; on the other hand, the shift was quite radical and possibly induced another fixed image with a different content. What stands to the fore is the presenter’s image of the other, which clearly determines her subjective position. Further suggestions supplied by the group (e.g. to compliment the patient; to invite her to speak about her dead husband; to encourage her to be more active in rebuilding a new life) served as cues for the presenter to deepen her understanding of the patient.

However, this changed perspective (from irritation to compassion) did not acknowledge the presenter’s more complex and ambivalent feelings about the situation. When an animator suggested to the presenter to share her concerns with the patient, this ambivalence particularly came to the fore. A renewed flow of irritation was triggered in the presenter, which indicates that her shift in perspective did not address the dimension of symbolic functioning. She reported “not knowing how much she wanted to share with her [the patient],” “not wanting to invest in *that* person,” and eventually remarked that “she [the patient] just seriously pissed her off.” She resolutely concluded that there are only two options: “either their relationship must end, or something must change.” One animator’s further elaboration on positive aspects of this doctor-patient relationship (e.g. the fact that they are creating a bond; that the GP is adopting the right technique by playing the waiting game; that she might be the patient’s ‘antidepressant’) appeared to actually enhance the presenter’s ambivalent feelings. As she searched for words to verbalise this incongruity, the presenter re-counted her last meeting with the patient, adding a salient detail. Apparently, when the patient had gestured for further interaction (“Are you already leaving?”), the presenter had been thinking that she “just wanted one thing: to escape.” Since the presenter seemed to be unaware of her ambivalence, an animator reflects back the presenter’s comment by stating: “she finally acknowledged you and then you wanted to escape.” The presenter’s initial difficulty to notice the ambivalence she had just expressed might indicate that she was surprised by her own words. At this point, the presenter appeared to be confronted with the otherness in herself, with forces that determine the situation on an unconscious level, or put differently: with her subjective dividedness. By acknowledging her tendency to escape from the patient, the presenter articulated her subjective implication in (the difficulties that characterise) the situation. This acknowledgement of the ambivalence she is confronted with (wanting to approach the patient, while also wanting to escape from her) contrasts sharply with her previous conscious conviction of wanting to develop a bond with the patient.

As this multiplicity of subjective positions was articulated, the presenter took up a more reflective stance, and gained a different perspective on the position she had been occupying in relation to the patient. The group discussion carried on for a little while. In response to one group member’s recapitulation of the discussion, criticizing the lack of exploration of the patient’s actual suffering, one animator emphasized having been impressed by the presenter’s sensitivity to the patient’s affectivity. With this intervention, she redefined the GP’s role as the carrier of a wide range of the patient’s emotions. The final minutes of the discussion were devoted to one group member’s suggestion to introduce some humour into their relationship and to be more playful with the patient.

The case follow-up one month later underscored the presenter’s altered subjective position, which impacted upon the doctor-patient interaction: “I saw her again and in fact, it was weird because the consultation was completely different. Normally it’s quite tense and we don’t succeed in having a real exchange. (…) Now, we’ve been able to have some sort of exchange and, in the end, it was interesting. It was the only time we had a real exchange; for once, it was pleasant. I think the dynamic has changed a little bit, so that’s good, she opened her heart to me, and well, that’s nice.” This follow-up was distinctively positive (e.g. “interesting,” “pleasant,” “nice”). The presenter’s discourse focused on their bond (e.g. “the consultation,” “the dynamic,” “we”) and also included reflective elements on the situation (e.g. referring to the “dynamic” of the interaction, making a comparison with their previous interactions). Remarkably, the presenter appeared to interpret the situation as if the patient had changed (e.g., “she spoke to me about her husband,” “she opened her heart”), which indicates that she is not entirely aware of her own altered position.

## Discussion

In order to illuminate the process of change in Balint group work, we analysed two case presentations and their subsequent group discussion. We concluded that Balint groups can be considered as a milieu in which GPs, who may be struggling with particular cases, can explore different angles from which these situations can be viewed. Balint group discussions often give rise to reflection that allows the presenters to take into account their subjective position in the relationship with the patient. First of all, the presenters’ willingness to present a case (in combination with their experience with Balint group work) can be seen as an indication of their readiness to put their perspective into question. Moreover, we believe that the shift from imaginary to symbolic relations is stimulated by the format of the group work. By stimulating free associative speech, the format encourages the presenters to (a) recognize aspects of their own subjectivity that don’t fit their ego; (b) acknowledge aspects of the otherness of the other that didn’t fit with the initial image of the patient; (c) transform their understanding of the problem they are struggling with. The shift towards the symbolic mode of relating to the other is stimulated by responses and interventions of the group members and animators. By asking questions and by articulating ideas, associations, images and emotions that are evoked during the discussion, group members and animators actively encourage the presenter to explore different subject positions. In the two cases outlined above, this shift from an imaginary to a symbolic mode of relating to the patient was observed. In both cases, the presenters appeared to be stuck in a fixed image of a situation (i.e., a chaotic situation that ended with the patient ‘dismissing’ the GP; an irritating patient who was difficult to approach). By verbalising the situation, as well as by interacting with the group, a more heterogenic range of subject positions was articulated. In the first case, the predominant focus on the complexity of the situation was extended with an exploration of the doctor-patient relationship. The presenter was able to take some distance from her spontaneous use of the employer-employee metaphor in depicting the relation with the patient, and to acknowledge the affective charge the situation induced. In the second case, the alternating focus on different patient characteristics prompted the presenter to acknowledge her ambivalent attitude.

Both cases demonstrated the *co*-*constructional* aspect of building and rebuilding a perspective with regard to a situation with a patient. The actual ‘change’ that takes place depends on the group’s interventions as well as the presenter’s capacity to take up cues for elaboration. In both groups, various interventions were administered, including challenging the presenter’s perspective, providing additional view points and encouraging reflection on unconscious dynamics that may influence the situation. Focusing on these dynamics, a Balint group meeting can be described as a continuous back and forth movement between providing space for the presenter to elaborate on questions, comments and suggestions, and the active introduction of new perspectives by the group. Depending on the presenter’s capacity to take up cues for elaboration, the subject positions that determine the GP’s interaction can be opened up. In the first case presentation, for instance, the presenter appeared to be unable to take up certain cues offered by the group (e.g. the meaning of lending money *only* to this patient), which indicates that she only partially recognized the symbolic dimension of her relation with the patient. In the second case presentation, the actual ‘change’ or the effect on the presenter’s subjective position is more clearly articulated. Here, the presenter’s shift from irritation to compassion seem to stir the initial images of the situation. Whereas, before the discussion, the presenter seemed to understand her difficulty in a rather one-dimensional way, the confrontation with her ambivalent stance disrupted this image. Exploring the right balance between confronting participants with unexplored perspectives on the one hand and respecting their defences on the other hand was found to be present in each of the groups. Moreover, in all four groups, members continually reported having been inspired by their peers’ presentations and by the group discussions, even during the meetings in which they had not presented a case.

Although Balint groups are not meant to be *therapeutic* groups, Balint group work can, to a certain extent, have a therapeutic effect [11,28]. In this context, we believe that the mere provision of “a space in which positions can be voiced and counter-positions assigned without considerations of ‘how’ realistic they are and without them being restrained by everyday rules of politeness” (2003: 547) [26] is crucial. The creation of such a reflective space is one of the elements that makes Balint group work quite unique. As formulated by Elder and Samuel (1987: 1) [29], Balint groups are expected to enable “a freeing from within range of personal reactions, rather than an imitative addition from without.” Change is said to lay in ‘the act of saying’ [15]. Along this way, members may be surprised by what unfolds. Similar to what occurs in a psychotherapeutic context, ‘change’ may become apparent by participants’ enhanced ability to adopt a wider range of discourses on the same theme, hold more complex views, and accept the perspectives of others [25]. In Balint group meetings, the aim is not to find the ‘true’ or ‘correct’ image of a situation (as such an image does not exist), nor is it to search for concrete ‘solutions’, but rather to open up the range of perspectives from which the situation can be viewed. Doing so might unlock blocked situations.

There are limitations to the present study. Because of our intention to analyze sessions on a detailed level, we were restricted to discussing only two cases. Nevertheless, examining more case presentations or studying group members’ change in discourse over several consecutive sessions (or even over several years of participation) could facilitate further understanding of the type of change members go through. Finally, while non-verbal group dynamics may also play a role in Balint group work, in this study we focused mainly on language, i.e. the presenter’s discourse and verbal group interactions. Indeed, in Lacan’s theory, verbal material comprises the essential structure around which meaning is constituted [30].

## Conclusion

For this study, we started from the observation that GP’s subjectivity plays an important role in their everyday work. By describing the difficulties GPs presented in Balint groups and the related (subjective) issues that were illuminated, we illustrated the way subjectivity can be present in their practice. Moreover, we threw light on the type of change Balint group participation allows for and on the way this is achieved. Hence, this study pointed out the potential usefulness of Balint group work with regard to GPs’ subjectivity as well as the possible benefit to the doctor-patient relationship.

## Abbreviations

GP: General practitioner.

## Competing interests

The authors declare that they have no competing interests.

## Authors’ contributions

KV and SV designed this study. KV gathered the data: she contacted the Balint groups, observed the Balint group meetings, audio-recorded them, and made notes about observations and impressions during and after the meetings. KV did the initial coding of the transcripts. KV, SV, VD, RI, RM and JD formed the research group: they repeatedly discussed the coding, as well as the theoretical concepts that ultimately guided the analysis. KV drafted the manuscript, which was extensively commented upon and rewritten by SV. All authors read and approved the final manuscript.

## Pre-publication history

The pre-publication history for this paper can be accessed here:

http://www.biomedcentral.com/1471-2296/15/49/prepub
